# Dyslipidemia and 10-year diabetes incidence in Japanese people: Population-based Panasonic cohort study 9

**DOI:** 10.3389/fendo.2022.957728

**Published:** 2022-08-05

**Authors:** Genki Kobayashi, Hiroshi Okada, Masahide Hamaguchi, Kazushiro Kurogi, Hiroaki Murata, Masato Ito, Michiaki Fukui

**Affiliations:** ^1^ Department of Endocrinology and Metabolism, Kyoto Prefectural University of Medicine, Graduate School of Medical Science, Kyoto, Japan; ^2^ Department of Diabetes and Endocrinology, Matsushita Memorial Hospital, Moriguchi, Japan; ^3^ Department of Health Care Center, Panasonic Health Insurance Organization, Moriguchi, Japan; ^4^ Department of Orthopaedic Surgery, Matsushita Memorial Hospital, Moriguchi, Japan

**Keywords:** incident diabetes, LDL/HDL ratio, LDL cholesterol, HDL cholesterol, cohort study

## Abstract

Low-density lipoprotein (LDL) cholesterol, high-density lipoprotein (HDL) cholesterol, and LDL/HDL ratio have been associated with new-onset diabetes; however, their cut-off levels have not been determined. We clarified the association between dyslipidemia and the incidence of diabetes. People who underwent a health checkup under a program conducted by Panasonic Corporation from 2008 to 2018 were included. In total, 87,570 participants were included, of whom 5,110 developed type 2 diabetes. Cox regression analyses and time-dependent receiver operating characteristic (ROC) curves were used to evaluate the association between LDL cholesterol, HDL cholesterol, or LDL/HDL ratio and incident diabetes and to identify the cut-off values for incident diabetes. Multivariate analysis showed that LDL cholesterol, HDL cholesterol, and LDL/HDL ratio were significantly associated with the risk of incident type 2 diabetes. Further, the area under the ROC curve and optimized cut-off values for LDL cholesterol, HDL cholesterol, and LDL/HDL ratio for incident type 2 diabetes at 10 years were 0.613 and 124 mg/dl, 0.640 and 54 mg/dl, and 0.662 and 2.4 mg/dl, respectively. The LDL/HDL ratio with a cut-off value of 2.4 was a better predictor of incident diabetes within 10 years than LDL and HDL cholesterol.

## Introduction

The number of patients with diabetes has increased in recent years. Without effective countermeasures, the diabetic population is projected to increase to 783 million by 2045 (1), and treatment costs associated with diabetes care will continue to increase. Therefore, the prevention of diabetes is extremely important.

Both dyslipidemia and diabetes are risk factors for atherosclerosis and increased vascular complications that may lead to mortality, such as cardiovascular disease (2, 3). Remarkably, diabetes and dyslipidemia frequently occur together (4). Previous reports have shown that dyslipidemia sometimes precedes the onset of hyperglycemia (5, 6), and low-density lipoprotein (LDL) cholesterol and high-density lipoprotein (HDL) cholesterol are also associated with new-onset diabetes (7). Recently, it has been reported that the LDL/HDL ratio is a more robust indicator of some diseases because it is based on two items instead of only one (7, 8). For instance, previous studies have shown that the LDL/HDL ratio is an indicator of cardiovascular disease (9), stroke (10), non-alcoholic fatty liver disease (11), microalbuminuria (12), and insulin resistance (13). Moreover, recent retrospective studies have suggested that higher LDL/HDL ratios are associated with an increased risk of incident diabetes (7). However, the cut-off values for LDL cholesterol, HDL cholesterol, and LDL/HDL ratio for predicting the incidence of diabetes are unknown. Moreover, the best predictor of diabetes has not yet been reported. Therefore, we conducted this retrospective cohort study to evaluate the association between dyslipidemia and a 10-year incidence of diabetes and to determine the cut-off values of LDL cholesterol, HDL cholesterol, and LDL/HDL ratio to indicate incident type 2 diabetes.

## Materials and methods

### Study population and study design

This long-term cohort study included participants from a medical health checkup program conducted by the Panasonic Corporation in Osaka, Japan. This program improved public health through the early detection of chronic diseases, including metabolic disorders, and by evaluating potential risk factors. All employees participated in this annual medical checkup. We used data collected between 2008 and 2018 from the database of the Panasonic Cohort Study.

Blood samples were obtained after fasting for >10 h. Weight and height were recorded using an automatic weight and height meter. The baseline characteristics were assessed using a self-administered questionnaire that was previously standardized and validated (14). Participants were categorized as nonsmokers, past smokers, or current smokers. Participants were also grouped into three levels of eating speed: fast, normal, and slow. The eating rate was determined using a questionnaire. The participants were asked about their breakfast habits. Participants who regularly engaged in any sport at least twice per week were defined as regular exercisers. Type 2 diabetes was defined as a fasting plasma glucose level of ≥126 mg/dl, a self-reported history of diabetes, and the use of anti-diabetic drugs.

This study was approved by the local ethics committee of the Panasonic Health Insurance Organization (approval number: 2021-001) and was conducted in accordance with the principles of the Declaration of Helsinki.

### Exclusion criteria

**Figure 1:**
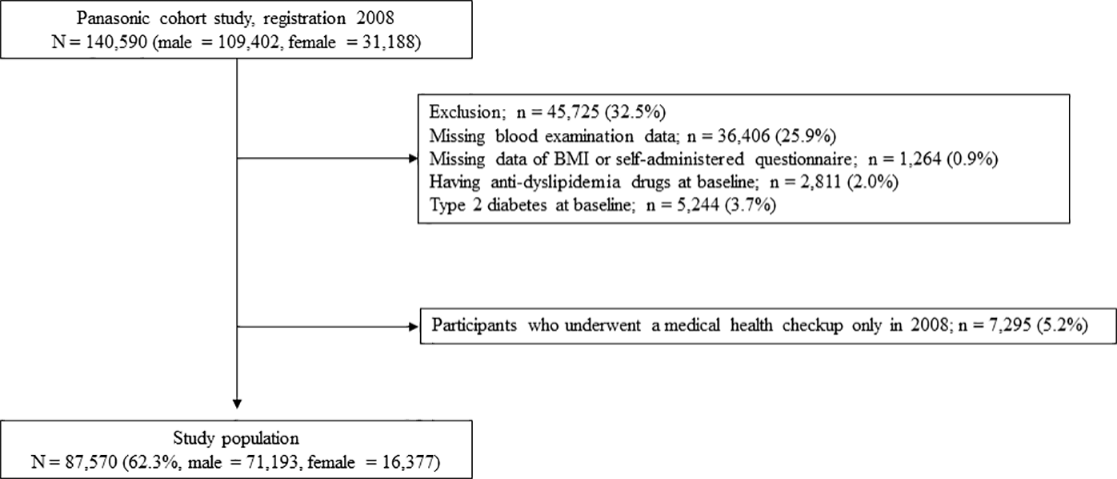
Study flow diagram for the registration of participants.

**Figure 1** shows the study flow diagram for the registration of participants. In total, 140,590 employees underwent medical health checkups in 2008. Participants who did not undergo a blood examination in 2008 (n = 36,406), participants with missing data (BMI or self-administered questionnaires) (n = 1,264), those who were taking anti-dyslipidemic drugs at baseline (n = 2,811), those with diabetes at baseline (N = 5,244), and those who had undergone a medical health checkup only in 2008 (n = 7,295) were excluded from the study.

### Statistical analyses

The means and frequencies of potential confounding variables were calculated. The Student’s t-test and the chi-square test were used to assess the differences in general characteristics at baseline according to the incidence of type 2 diabetes at the 10-year follow-up. The association between LDL cholesterol, HDL cholesterol, or LDL/HDL ratio and incident type 2 diabetes was evaluated by Cox regression analyses using multivariate models. The covariates included in the multivariate models were the factors associated with incident type 2 diabetes. The multivariate model was adjusted for age, sex, BMI, systolic blood pressure, triglycerides, uric acid, fasting plasma glucose, smoking status, eating speed, skipping breakfast, and exercise habits. LDL cholesterol and HDL cholesterol levels were added to Model 1. The LDL/HDL ratio was added to model 2 instead of LDL cholesterol and HDL cholesterol. To evaluate the predictive performance of LDL cholesterol, HDL cholesterol, and LDL/HDL ratio, we employed time-dependent ROC curves for censored survival data and the AUC as criteria. We compared the AUC of LDL cholesterol, HDL cholesterol, and LDL/HDL ratio with the Bootstrap method. We assessed the optimal cut-off values of LDL cholesterol, HDL cholesterol, and LDL/HDL ratio for incident type 2 diabetes in all patients, patients with BMI <25 kg/m^2^, and patients with BMI ≥25 kg/m^2^. All continuous variables are presented as the mean ± SD or absolute numbers. Differences were considered statistically significant at P <0.05. The associations are presented as HRs with a 95% CI. Statistical analyses were conducted using JMP software, version 12 (SAS Institute, Cary, NC, USA).

## Results

**Table 1** shows the baseline characteristics of the participants. A total of 5,110 participants developed type 2 diabetes during the study period. Biochemical tests showed significantly higher levels of LDL cholesterol and lower levels of HDL cholesterol in the population with incident diabetes than in those without incident diabetes. **Table 2** shows the unadjusted and adjusted hazard ratios (HRs) for incident type 2 diabetes. Multivariate analysis showed that LDL cholesterol (HR; 1.02 [95% confidence interval (CI): 1.01–1.03]), HDL cholesterol (HR; 0.89 [95% CI: 0.87–0.91]), and LDL/HDL ratio (HR; 1.19 [95% CI: 1.16–1.23]) were significantly associated with the risk of incident type 2 diabetes.

**Table 1:** Characteristics of Panasonic Cohort Study participants at baseline by diabetes incidence during the 10-year follow-up period.

	ALL	Diabetes incidence (−)	Diabetes incidence (+)	P-value
N	87,570	82,460	5,110	–
Age (y)	44.7 (8.1)	44.5 (8.2)	46.7 (6.5)	<0.0001
Sex (male/female) (%)	71,193 /16,377 (81.3/18.7)	66,427/16,033 (80.6/19.4)	4,766/344 (93.3/6.7)	<0.0001
Body mass index (kg/m^2^)	23.0 (3.3)	22.8 (3.2)	25.6 (4.0)	<0.0001
Systolic blood pressure (mmHg)	119.1 (14.6)	118.7 (14.5)	126.3 (15.8)	<0.0001
Diastolic blood pressure (mmHg)	74.5 (11.0)	74.2 (10.9)	79.8 (11.2)	<0.0001
LDL cholesterol (mg/dl)	124.5 (31.2)	123.8 (31.0)	135.2 (32.3)	<0.0001
HDL cholesterol (mg/dl)	59.8 (15.1)	60.2 (15.1)	53.3 (13.7)	<0.0001
Triglycerides (mg/dl)	112.8 (86.1)	110.2 (83.5)	155.6 (112.8)	<0.0001
Fasting plasma glucose (mg/dl)	93.3 (9.2)	92.5 (8.5)	106.2 (10.5)	<0.0001
Uric acid (mg/dl)	5.8 (1.4)	5.8 (1.4)	6.4 (1.3)	<0.0001
Smoking (none/past/current) (%)	44,727/11,454/31,389 (51.1/13.1/35.8)	42,688/10,740/29,032 (51.8/13.0/35.2)	2,039/714/2,357 (39.9/14.0/46.1)	<0.0001
Eating speed (fast/normal/slow) (%)	28,880/52,358/6,332 (33.0/59.8/7.2)	26,717/49,620/6,123 (32.4/60.2/7.4)	2,163/2,738/209 (42.3/53.6/4.1)	<0.0001
Skipping breakfast (+/−) (%)	18,557/69,013 (21.2/78.8)	17,280/65,180 (21.0/79.0)	1,277/3,833 (25.0/75.0)	<0.0001
Physical exercise (+/−) (%)	14,661/72,909 (16.7/83.3)	13,781/68,679 (16.7/83.3)	880/4,230 (17.2/82.8)	0.34

Data are presented as mean (standard deviation, or percentage) or absolute number.

LDL, low-density lipoprotein; HDL, high-density lipoprotein.

Chi-square test were used to assess the differences between diabetes incidence (−) and (+).

**Table 2:** Unadjusted and adjusted hazard ratios for incidence of diabetes during the 10-year follow-up period.

	Crude	Model 1	Model 2
	HR (95% CI)	HR (95% CI)	HR (95% CI)
Age (per 10 years)	2.06 (1.98–2.15)	1.35 (1.28–1.41)	1.34 (1.28–1.41)
Sex (ref: female)	3.04 (2.73–3.39)	0.88 (0.78–0.9998)	0.92 (0.81–1.04)
Body mass index (per 1 kg/m^2^)	1.20 (1.19–1.21)	1.10 (1.09–1.11)	1.10 (1.09–1.11)
Systolic blood pressure (per 10 mmHg)	1.39 (1.36–1.41)	1.03 (1.01–1.05)	1.03 (1.01–1.05)
Low-density lipoprotein cholesterol (per 10 mg/dl)	1.12 (1.11–1.13)	1.02 (1.01–1.03)	–
High-density lipoprotein cholesterol (per 10 mg/dl)	0.71 (0.69–0.72)	0.89 (0.87–0.91)	–
LDL/HDL ratio	1.69 (1.65–1.73)	–	1.19 (1.16–1.23)
Triglycerides (per 10 mg/dl)	1.02 (1.020–1.022)	1.005 (1.002–1.007)	1.006 (1.004–1.008)
Uric acid (per 1 mg/dl)	1.31 (1.28–1.33)	0.99 (0.97–1.02)	0.99 (0.97–1.01)
Fasting plasma glucose (per 10 mg/dl)	4.38 (4.27–4.50)	3.86 (3.75–3.98)	3.85 (3.74–3.97)
Smoking (past) (ref: none)	1.39 (1.28–1.51)	1.05 (0.96–1.14)	1.04 (0.95–1.14)
Smoking (current) (ref: none)	1.69 (1.59–1.79)	1.56 (1.47–1.66)	1.57 (1.47–1.67)
Eating speed (slow) (ref: normal)	0.64 (0.55–0.73)	0.91 (0.78–1.04)	0.90 (0.78–1.04)
Eating speed (fast) (ref: normal)	1.40 (1.33–1.48)	1.09 (1.03–1.15)	1.09 (1.03–1.16)
Skipping breakfast (yes) (ref: no)	1.20 (1.12–1.27)	1.06 (0.99–1.13)	1.05 (0.98–1.12)
Physical exercise (yes) (ref: no)	1.09 (1.01–1.17)	0.98 (0.91–1.06)	0.98 (0.91–1.05)

LDL, low-density lipoprotein; HDL, high-density lipoprotein.

**Figure 2:**
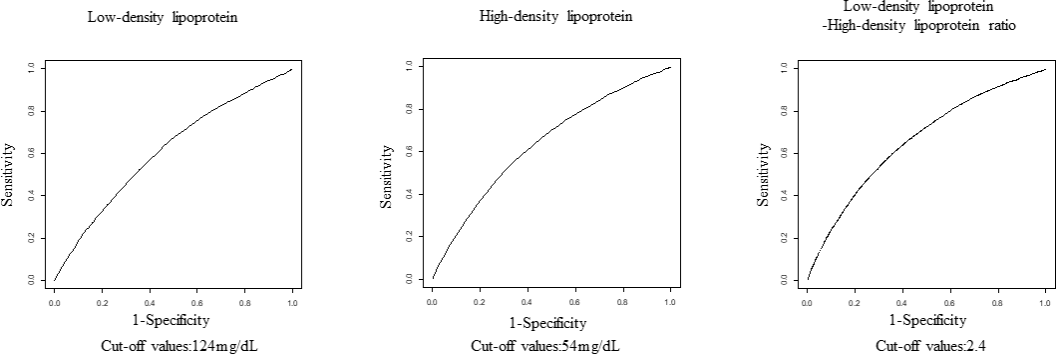
The receiver operating characteristic curve showing the ability of LDL, HDL, and LDL/HDL ratio to determine the incidence of diabetes after 10 years of follow-up. LDL, low-density lipoprotein; HDL, high-density lipoprotein.

Time-dependent receiver operating characteristic (ROC) curve analysis revealed the area under the curve (AUC) at 10 years. The AUC and optimized cut-off values for LDL cholesterol, HDL cholesterol, and LDL/HDL ratio for incident type 2 diabetes at 10 years were 0.613 and 124 mg/dl, 0.640 and 54 mg/dl, and 0.662 and 2.4 mg/dl, respectively. The AUC of the LDL/HDL ratio was higher than that of LDL cholesterol (difference value; 0.05, 95% CI; 0.044 to 0.055, P <0.0001) or HDL cholesterol (difference value; 0.019, 95% CI; 0.013 to 0.024, P <0.0001). **Figure 2** and **Table 3** show the time-dependent ROC curve that indicates the ability of LDL cholesterol, HDL cholesterol, and LDL/HDL ratio for predicting the incidence of diabetes. The sensitivity and specificity of LDL cholesterol, HDL cholesterol, and LDL/HDL ratio to predict the incidence of diabetes were 64.2% and 53.3%, 57.7% and 63.7%, and 60.7% and 63.7%, respectively. The AUC and optimal cut-off values were similar in all patients, patients with a body mass index (BMI) <25 kg/m^2^, and patients with a BMI ≥25 kg/m^2^ (**Table 3**).

**Table 3:** The area under the curve and optimal cut-off values.

Whole participants	AUC	Sensitivity	Specificity	Cut-off value
Low-density lipoprotein cholesterol	0.613	64.2%	53.3%	124 mg/dl
High-density lipoprotein cholesterol	0.640	57.7%	63.7%	54 mg/dl
LDL/HDL ratio	0.662	60.7%	63.7%	2.4
**Body mass index < 25 kg/m^2^**	**AUC**	**Sensitivity**	**Specificity**	**Cut-off value**
Low-density lipoprotein cholesterol	0.595	57.6%	57.7%	124 mg/dl
High-density lipoprotein cholesterol	0.600	64.2%	50.6%	61 mg/dl
LDL/HDL ratio	0.624	54.4%	64.4%	2.3
**Body mass index < 25 kg/m^2^**	**AUC**	**Sensitivity**	**Specificity**	**Cut-off value**
Low-density lipoprotein cholesterol	0.553	62.0%	46.0%	130 mg/dl
High-density lipoprotein cholesterol	0.577	64.0%	48.7%	52 mg/dl
LDL/HDL ratio	0.591	57.5%	56.6%	2.8

LDL, low-density lipoprotein; HDL, high-density lipoprotein.

## Discussion

Our major findings were as follows: (1) we obtained cut-off values of 124 mg/dl, 54 mg/dl, and 2.4 mg/dl for LDL cholesterol, HDL cholesterol, and LDL/HDL ratio, respectively, for incident type 2 diabetes within 10 years, and (2) the LDL/HDL ratio was more predictive of incident diabetes within 10 years than LDL and HDL cholesterol.

Dyslipidemia in type 2 diabetes is characterized by high triglyceride or LDL cholesterol levels and low HDL levels (15, 16). Previous reports have shown that both LDL and HDL cholesterol are associated with the risk of developing diabetes (5, 6, 17). Previous studies have reported that lipid ratios are more sensitive than individual lipid indices (e.g., HDL cholesterol, LDL cholesterol, and triglycerides) for the diagnosis and prevention of coronary atherosclerotic disease in patients with type 2 diabetes (15, 18). Moreover, other studies have shown that the LDL/HDL ratio is a valid predictor of insulin resistance in healthy adults (19) and that this parameter is associated with incident diabetes in patients with hypertension (20). It has also been shown that the incidence of diabetes increases as the LDL/HDL ratio increases (7). However, these studies have some limitations; they are cross-sectional studies (19, 20) or have follow-up periods of only a few years (7). Moreover, the use of anti-dyslipidemic drugs, including statins, which could affect the incidence of diabetes (21, 22), was not considered in a previous study (7). Furthermore, the cut-off levels for LDL cholesterol, HDL cholesterol, and LDL/HDL ratio that are most associated with incident diabetes have not been determined.

It has been reported that an LDL/HDL ratio of <2 inhibits plaque growth and regression due to atherosclerosis (23). Another study has shown that an LDL/HDL ratio of <2.5 is useful for screening atherosclerosis (24). These cut-off values are consistent with our results. We assessed the optimal cut-off values of LDL cholesterol, HDL cholesterol, and LDL/HDL ratio for incident type 2 diabetes in patients with BMI <25 kg/m^2^, and in patients with BMI ≥25 kg/m^2^, which is the definition of obesity in Japan, because it has been reported that obesity could modulate the relationship between dyslipidemia and diabetes due to insulin resistance and relative insulin deficiency, and the risk factors of incident diabetes vary with BMI in the Japanese population (25). The AUC and optimal cut-off values in this study were similar for both obese and non-obese patients.

The relationship between dyslipidemia and diabetes may be as follows: LDL cholesterol decreases the expression of cyclin B1 in pancreatic β-cells and inhibits insulin secretion mediated by the LDL receptor, resulting in insulin resistance (5, 7, 26–28). In contrast, HDL cholesterol improves insulin resistance by inhibiting β-cell apoptosis, promoting insulin secretion, and inhibiting the action of LDL cholesterol (5, 7, 26–28). Additionally, diabetes and insulin resistance can lead to high triglyceride levels, resulting in decreased HDL cholesterol levels (29, 30).

Interestingly, a previous study has reported a U-shaped association between the LDL/HDL ratio and all-cause mortality in elderly hypertensive patients (31). The reasons why not only higher LDL/HDL ratios but also lower LDL/HDL ratios were associated with higher all-cause mortality are thought to be as follows. The lower LDL/HDL ratio was caused by higher HDL cholesterol levels. Bowe et al. (32) reported that a higher HDL cholesterol level was associated with increased mortality. HDL cholesterol loses its protective effect at higher levels (33). No U-shaped association between the LDL/HDL ratio and incident type 2 diabetes was not found in this study (data not shown).

The strength of our study stem from its large sample size and long follow-up duration because this cohort study was conducted using health examinations of the corporation. Furthermore, patients taking anti-dyslipidemia drugs that could affect lipid profiles were excluded. However, one limitation of this study was that only relatively young Japanese subjects were included. Therefore, it is unclear whether the results of this study can be generalized to other ethnic categories and age groups.

We determined the cut-off levels of LDL cholesterol, HDL cholesterol, and LDL/HDL ratio for 10-year diabetes incidence. Additionally, we found that LDL/HDL ratio was more useful than LDL and HDL cholesterol in predicting incident diabetes within 10 years. Therefore, it is important to focus on LDL cholesterol, HDL cholesterol, and, especially, LDL/HDL ratio to assess the risk of diabetes, and if screening is positive, the patients should be carefully monitored and early intervention should be considered, including diet and exercise therapy, to prevent the incidence of diabetes.

## Data availability statement

The original contributions presented in the study are included in the article/supplementary material. Further inquiries can be directed to the corresponding author.

## Ethics statement

The studies involving human participants were reviewed and approved by the local ethics committee of the Panasonic Health Insurance Organization. Written informed consent for participation was not required for this study in accordance with the national legislation and the institutional requirements.

## Author contributions

GK wrote the manuscript. MH, KK, and HM contributed to the discussion. HO and MI researched the data and contributed to the conception and discussion. HO and MF reviewed and edited the manuscript. All authors listed have made a substantial, direct, and intellectual contribution to the work and approved it for publication.

## Conflict of interest

HO received personal fees from MSD K.K., Mitsubishi Tanabe Pharma Corporation, Sumitomo Dainippon Pharma Co., Ltd., Novo Nordisk Pharma Ltd., Daiichi Sankyo Co., Ltd, Eli Lilly Japan K.K, Kyowa Hakko Kirin Company Ltd, Kissei Pharmaceutical Co., Ltd, Kowa Pharmaceutical Co., Ltd, Ono Pharmaceutical Co., Ltd. and Sanofi K.K. M.H. received grants from Ono Pharma Co. Ltd., AstraZeneca K.K., Oishi Kenko Inc., Yamada Bee Farm and received personal fees from Sumitomo Dainippon Pharma Co., Ltd., AstraZeneca K.K., Ono Pharma Co. Ltd., Eli Lilly, Japan, Daiichi Sankyo Co. Ltd., Mitsubishi Tanabe Pharma Corp., Sanofi K.K., K.K., Kowa Pharma Co. Ltd., outside the submitted work. MF received grants from Ono Pharma Co. Ltd., Oishi Kenko inc., Yamada Bee Farm, Astellas Pharma Inc., Mitsubishi Tanabe Pharma Corp., Nippon Boehringer Ingelheim Co. Ltd., MSD K.K., Kissei Pharma Co. Ltd., Daiichi Sankyo Co. Ltd., Sanwa Kagagu Kenkyusho Co., Ltd., Sanofi K.K., Takeda Pharma Co. Ltd., Kyowa Kirin Co., Ltd., Sumitomo Dainippon Pharma Co., Ltd., Terumo Corp., Tejin Pharma Ltd., Novo Nordisk Pharma Ltd., Eli Lilly, Japan, K.K., Taisho Pharma Co., Ltd., Abbott Japan Co. Ltd., Nippon Chemiphar Co., Ltd., Kowa Pharma Co. Ltd. and Johnson & Johnson K.K. Medical Co. and received personal fees from Abbott Japan Co. Ltd., Kissei Pharma Co., Ltd., Sumitomo Dainippon Pharma Co. Ltd., Mitsubishi Tanabe Pharma Corp., Daiichi Sankyo Co. Ltd., Sanofi K.K., Astellas Pharma Inc., MSD K.K., Kyowa Kirin Co. Ltd., Taisho Pharma Co., Ltd., Kowa Pharma Co. Ltd., Mochida Pharma Co. Ltd., Novo Nordisk Pharma Ltd., Ono Pharma Co. Ltd., Sanwa Kagaku Kenkyusho Co. Ltd., Eli Lilly Japan K.K., Takeda Pharma Co. Ltd., Bayer Yakuhin, Ltd., AstraZeneca K.K., Nippon Boehringer Ingelheim Co., Ltd., Teijin Pharma Ltd., Medtronic Japan Co. Ltd., Arkray Inc. and Nipro Corp. outside the submitted work.

The remaining authors declare that the research was conducted in the absence of any commercial or financial relationships that could be construed as a potential conflict of interest.

## Publisher’s note

All claims expressed in this article are solely those of the authors and do not necessarily represent those of their affiliated organizations, or those of the publisher, the editors and the reviewers. Any product that may be evaluated in this article, or claim that may be made by its manufacturer, is not guaranteed or endorsed by the publisher.
