# Erratum: Dyslipidemia and 10-year diabetes incidence in Japanese people: Population-based Panasonic cohort study 9

**DOI:** 10.3389/fendo.2022.1042407

**Published:** 2022-09-29

**Authors:** Frontiers Production Office

**Affiliations:** Frontiers Media SA, Lausanne, Switzerland

**Keywords:** incident diabetes, LDL/HDL ratio, LDL cholesterol, HDL cholesterol, cohort study

Due to a production error, there was a mistake in **Table 3** as published. The third sub-heading within the table was incorrectly captured as “Body mass index < 25 kg/m2” instead of “Body mass index ≥ 25 kg/m2”.

The corrected **Table 3** appears below.

The publisher apologizes for this mistake.

The original version of this article has been updated.

**Table 3:** The area under the curve and optimal cut-off values.

Whole participants	AUC	Sensitivity	Specificity	Cut-off value
Low-density lipoprotein cholesterol	0.613	64.2%	53.3%	124 mg/dl
High-density lipoprotein cholesterol	0.640	57.7%	63.7%	54 mg/dl
LDL/HDL ratio	0.662	60.7%	63.7%	2.4
**Body mass index < 25 kg/m^2^**	**AUC**	**Sensitivity**	**Specificity**	**Cut-off value**
Low-density lipoprotein cholesterol	0.595	57.6%	57.7%	124 mg/dl
High-density lipoprotein cholesterol	0.600	64.2%	50.6%	61 mg/dl
LDL/HDL ratio	0.624	54.4%	64.4%	2.3
**Body mass index ≥ 25 kg/m^2^**	**AUC**	**Sensitivity**	**Specificity**	**Cut-off value**
Low-density lipoprotein cholesterol	0.553	62.0%	46.0%	130 mg/dl
High-density lipoprotein cholesterol	0.577	64.0%	48.7%	52 mg/dl
LDL/HDL ratio	0.591	57.5%	56.6%	2.8

LDL, low-density lipoprotein; HDL, high-density lipoprotein.

